# Interactive anticancer effect of nanomicellar curcumin and galbanic acid combination therapy with some common chemotherapeutics in colon carcinoma cells

**Published:** 2019

**Authors:** Arash Jafari, Manouchehr Teymouri, Maryam Ebrahimi Nik, Azam Abbasi, Mehrdad Iranshahi, Mohammad Yahya Hanafi-Bojd, Mahmoud Reza Jafari

**Affiliations:** 1 *School of Medicine, Birjand University of Medical Sciences, Birjand, Iran. *; 2 *Natural Products and Medicinal Plants Research Center, North Khorasan University of Medical Sciences, Bojnurd, Iran.*; 3 *Nanotechnology Research Center, Pharmaceutical Technology Institute, Mashhad University of Medical Sciences, Mashhad, Iran.*; 4 *Biotechnology Research Center, Pharmaceutical Technology Institute, Mashhad University of Medical Sciences, Mashhad, Iran. *; 5 *Cellular and Molecular Research Center, Birjand University of Medical Sciences, Birjand, Iran.*

**Keywords:** Nanomicellar curcumin Galbanic acid, Doxil, Cisplatin, Combination therapy Synergism

## Abstract

**Objective:**

In the current investigation, we aimed to study the combined cytotoxicity of curcumin, as a nanomicellar formulation, and galbanic acid (Gal), dissolved in DMSO against the murine C26 and human Caco-2 colon carcinoma cells. Further, curcumin potential for cisplatin and doxorubicin (Dox) co-therapy was studied.

**Materials and Methods:**

The combined cytotoxic effect of these phytochemicals at varying dose ratios were examined using the MTT colorimetric assay. Moreover, the time-dependent toxicity of curcumin, cisplatin, Dox, and pegylated liposomal Dox (Doxil) was determined. The interactive anti-proliferative behavior of these compounds was examined using the CompuSyn software.

**Results:**

Nanomicellar curcumin showed considerable cytotoxicity in C26 cells 24 hr post-treatment. Co-treatment of cells with curcumin nanomicelles: Gal had a synergistic effect in C26 (at 10:1 molar ratio), and Caco-2 (at 1:5 molar ratio) cell lines in cell cultures. Nanomicellar curcumin showed strong and mild synergistic inhibitory effects in C26 cells when co-administered with Doxil and cisplatin, respectively.

**Conclusion:**

Curcumin nanomicelles and Gal had a synergistic effect in C26 and Caco-2 cell lines. It is speculated that nanomicellar curcumin shows synergistic cancer cell killing if administered 24-hr post-injection of Doxil and cisplatin.

## Introduction

Cancer chemotherapy has remained the most common medical intervention for treatment of cancers (Barenholz, 2012 ▶). The rationale behind cancer chemotherapy is that cancer cells are usually more sensitive to chemotherapeutics compared to normal tissues and if chemotherapeutics consumed at an appropriate dose schedule, they can manage to treat cancer (Shoham et al., 1970 ▶; Leibbrandt and Wolfgang, 1995 ▶). However, this scenario was shown to face various complications including the development of drug-resistant cancer cell populations and chemotherapeutic-related side effects (Salmon et al., 1989 ▶; Barenholz, 2012 ▶). In this regard, any agent that could enhance cancer cell growth inhibition and prevent normal tissues from off-targeted adverse effects of chemo-agents, could add to the therapeutic value of the chemotherapy regimen (Teymouri et al., 2018 ▶). 

Curcumin and galbanic acid are two shining examples of phytochemicals with prospects of improving anti-cancer responses of chemotherapy in the future (Kim et al., 2011 ▶; Teymouri et al., 2017 ▶). Curcumin is the major compound extracted from the rhizome of turmeric (*Curcuma longa*) and galbanic acid is a sesquiterpene coumarin mainly found in the genus *Ferula* (Apiaceae). Despite many problems for their therapeutic application (such as their poor pharmacokinetic profile and fast metabolism), the multi-faceted anti-cancer property of these agents has motivated many researchers to use them as a supplementation to the current cancer chemotherapy regimen (Hanafi-Bojd et al., 2016 ▶; Teymouri et al., 2017 ▶). Curcumin and galbanic acid are reported to exhibit pro-apoptotic activity in cancer cells, as well as anti- angiogenic, anti-inflammatory, hepato-protective and chemo-preventive activities, which could be beneficial for both treatment of cancer cells and tumors and protection of normal tissues (Hanafi-Bojd et al., 2011 ▶; Kim et al., 2011 ▶; Kasaian et al., 2013 ▶, 2015; Oh et al., 2015 ▶; Teymouri et al., 2017 ▶). 

Eliciting a synergistic response in treating cancer cells using drug combinations is regarded desirable as it evades the development of drug resistance and reduces the required dose of chemo-agents (Greco and Vicent, 2009 ▶; Hu and Zhang, 2012 ▶). Moreover, it avoids damage to normal tissues due to reduction of the required dose (Greco and Vicent, 2009 ▶). The therapy response varies and depends on many factors, including the cell type, choice of drug, relative dose ratio, sequence of drug treatment, and duration of drug exposure (Huq et al., 2014 ▶). 

In the current study, we applied different ratios of nanomicellar curcumin and galbanic acid in murine C26 and human Caco-2 colon carcinoma cells. Throughout the text, the terms “nanomicellar curcumin” and “nanocurcumin” are used interchangeably. The cytotoxicity of nanomicellar curcumin and galbanic acid was assessed in C26 and Caco-2 cells and the results were used to calculate the combined cytotoxicity of these compounds at varying dose ratios. The cytotoxicity of the nanomicellar curcumin and galbanic acid was tested at curcumin:galbanic acid dose ratios of 10:1, 5:1, 1:1, 1:5, and 1:10, where the figures 10-, 5-, 1- imply fold doses of the half-maximal inhibitory concentrations (IC_50_) of the agents in the series of the combination study. Moreover, the time-evolution anti-cancer effects of the nanocurcumin, cisplatin, doxorubicin (Dox) solution, and Doxil were examined. Finally, the nanocurcumin/Doxil and nanocurcumin/cisplatin combination therapy was studied. 

## Materials and Methods

**Materials**

Curcumin was purchased from Sami Labs Limited (Bengaluru, Karnataka, India). Nanomicellar curcumin was manufactured by Exir Nano Sina Company (Tehran, Iran). Purified galbanic acid was kindly provided by Prof. Mehrdad Iranshahi, School of Pharmacy, Mashhad University of Medical Sciences, Mashhad Iran, which was extracted as per a previously described method (Ahmadi et al., 2017 ▶). MTT (3-(4, 5-dimethylthiazol-2-yl)-2, 5diphenyltetrazolium bromide) was purchased from Promega (Madison, WI). All other reagents were of chemical grade. 

**Cell culture**

Murine C26 and human Caco-2 colon carcinoma cell lines were grown in RPMI1640 medium supplemented with 10% (v/v) heat-inactivated fetal calf serum (FCS), 2 mM L-glutamine, 100 IU/ml penicillin and 100 mg/ml streptomycin at 37^o^C under humidified atmosphere containing 5% CO_2_ in an incubator. The viable cells were counted at the beginning of the cell experiment using trypan blue-dye exclusion method (Strober, 2015 ▶). 

**Toxicity of nanomicellar curcumin and galbanic acid**

Prior to the experiment, galbanic acid (20 mg/ml) and curcumin powder (30 mg/ml) were dissolved in dimethyl sulfoxide (DMSO) on the day of cell treatment. The nanomicellar curcumin contained 7% w/v curcumin with a narrow size distribution (polydispersity index of 0.2) and mean diameter of 10 nm, according to dynamic light scattering (Ahmadi et al., 2018 ▶). 

For both cell lines, approximately 5×10^3^ viable cells/well were seeded into a flat-bottomed 96-well plate and incubated overnight in an incubator. At 24 hr, freshly prepared curcumin (30 mg/ml) and galbanic acid (20 mg/ml) as well as nanomicellar curcumin (30 mg/ml), were diluted serially in RPMI-FCS and added to the plate in eight replicates. The nanomicellar curcumin, which was miscible with aqueous phases, was readily diluted serially in the RPMI-FCS and added to the plate. The plate was incubated for further 48 hr and then, the medium was replaced with the same volume of the fresh medium mixed with an MTT solution (5 mg/ml; 9:1 v/v) (Hanafi-Bojd et al., 2015 ▶). At 4-hr post-incubation, the medium was removed and the cells were washed twice with phosphate-buffered saline (PBS). Subsequently, 0.2 ml of DMSO was added to the wells and mixed thoroughly to dissolve the produced formazan in each well. Finally, the absorbance was recorded at 550 nm with the reference absorbance at 660 nm by a Multiscan plus plate reader (Labsystems).

To study their combination effect, nanomicellar curcumin and galbanic acid were mixed together at varying ratios as follows: 10-fold concentration of curcumin IC_50_ dose to 1-fold concentration of galbanic acid IC_50_ dose (Cur/Gal 10:1), 5-fold concentration of curcumin IC_50_ dose to 1-fold concentration of galbanic acid IC_50_ dose (Cur/Gal 5:1), 1-fold-to-1-fold dose ratio (Cur/Gal 1:1), 1-fold-to-5-fold dose ratio (Cur/Gal 1:5) and 1-fold-to-10-fold dose ratio (Cur/Gal 1:10). From the mentioned mixed stock solutions, the cells were treated with the serial dilutions. 

**Nanomicellar curcumin/chemotherapeutics combination therapy**

The same MTT cytotoxicity assay was done in C26 cells for the nanomicellar curcumin, doxorubicin (Dox), pegylated liposomal doxorubicin (Doxil™) and cisplatin as described in “Toxicity of nanomicellar curcumin and galbanic acid”. For the combination therapy, the nanomicellar curcumin was applied in combination with Doxil at 1-to-10 and 10-to-1 mole ratios; and with cisplatin at 1-to-1 and 1-to-10 mole ratios. Other conditions were similar, including nearly 5×10^3 ^cells/well seeding, 48-hr drug treatment and 4-hr MTT exposure. 

**Time-course toxicity study**

The time-course cytotoxicity of the nanomicellar curcumin and the chemo-agents, i.e. doxorubicin (2 mg/ml), Doxil (2 mg/ml) and cisplatin (1mg/ml) was measured in C26 cells. For this, 2.5×10^3^, 5×10^3^ and 1×10^4^ C26 cells/well were seeded into 96-well plates to measure the cytotoxicity of 72-hr, 48-hr and 24-hr treatments, respectively. At 24 hr, 1×10^4^ cells/well was treated for 24 hr with serial dilutions of the nanomicellar curcumin and the chemo-agents. Similarly, 5×10^3^ and 2.5×10^3^ cells/well were treated with the drugs for 48 and 72hr, respectively. For the control, several wells were left out without drug treatment. At 24, 48 and 72 hr, the mentioned wells were treated with the MTT reagent for 2 hr and put into the subsequent processes as described above. 

**Statistical analysis**

The data analysis was performed using the freely available (http://www.combosyn.com/) software of CompuSyn. For this purpose, the absorbance data were first normalized for lowest and the highest values at 0 and 100 in the datasets with GraphPad Prism version 5 (GraphPad Software, San Diego, CA). The average of the normalized data was imported into CompuSyn software. To assess the drug-drug interaction with respect to the cytotoxic response, combination index (CI) and drug-reduced index (DRI) were tabulated and plotted. Finally, the statistical analysis was conducted using the GraphPad Prism. Two-tailed statistical analysis was carried out at a significance level of 0.05. In addition, one-way ANOVA and Newman–Keuls multiple comparison test were used as needed.

## Results

**Cytotoxicity of each individual agent**

**Figure 1:**
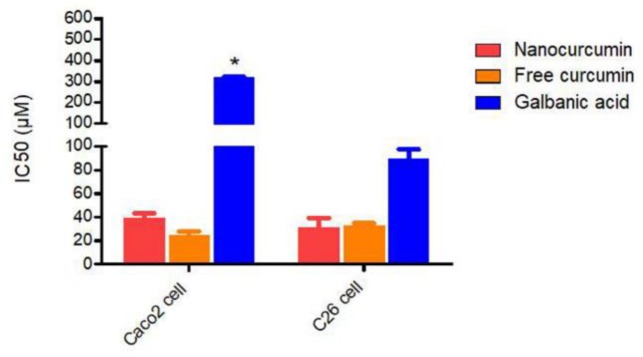
Half-maximal inhibitory concentrations (IC_50_) of curcumin, nanocurcumin and galbanic acid. Data are shown as mean±SD of three independent replicates (p<0.05)

Curcumin was found to be more cytotoxic in both C26 and Caco-2 cell lines compared to galbanic acid (Figure 1). While galbanic acid was not comparably effective in limiting cancer cell growth in Caco-2 cells as they were in C26 cells; however, curcumin exhibited similar cytotoxicity in both cell types. The cytotoxicity of the nanomicellar curcumin was slightly lower than that of curcumin. With respect to galbanic acid, the calculated IC_50_ values were about 3- and 7-fold higher than those of curcumin in C26 and Caco-2 cells, respectively.

**Figure 2:**
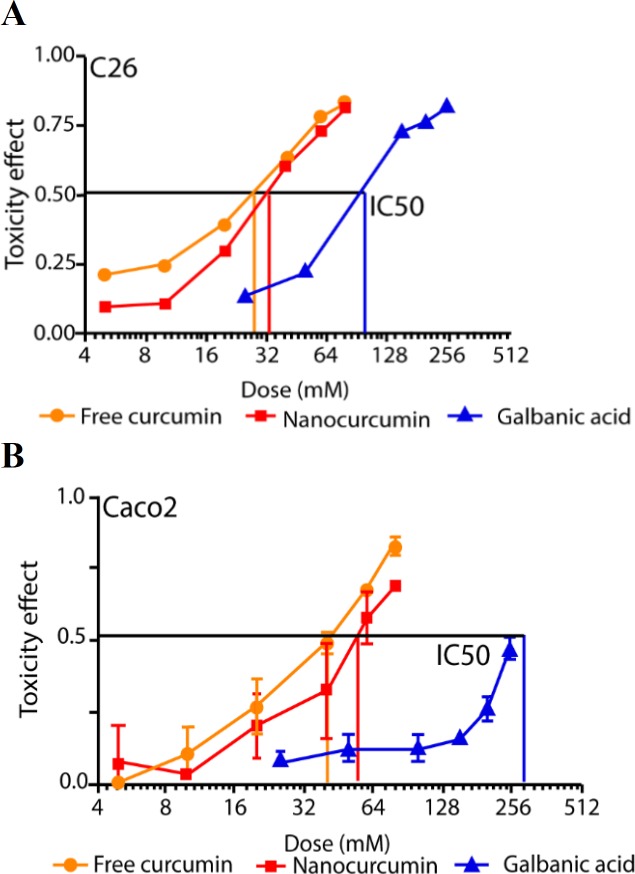
The dose-dependent cytotoxic trend of curcumin, nanocurcumin and galbanic acid in C26 cells (A) and Caco-2 cell (B). Data are shown as mean±SD of 8 replicates (p<0.05)

The cytotoxicity of these agents was dose-dependent as shown in Figure 2. In both cells, the anti-proliferative activity of galbanic acid started at much higher doses than those of curcumin powder and nanomicellar curcumin (p<0.05). Similar dose-responsive trends for both curcumin and nanomicellar curcumin were found with no significant difference in the toxicity of the compounds at varying doses. 

**Combined curcumin/galbanic acid toxicity**

**Figure 3:**
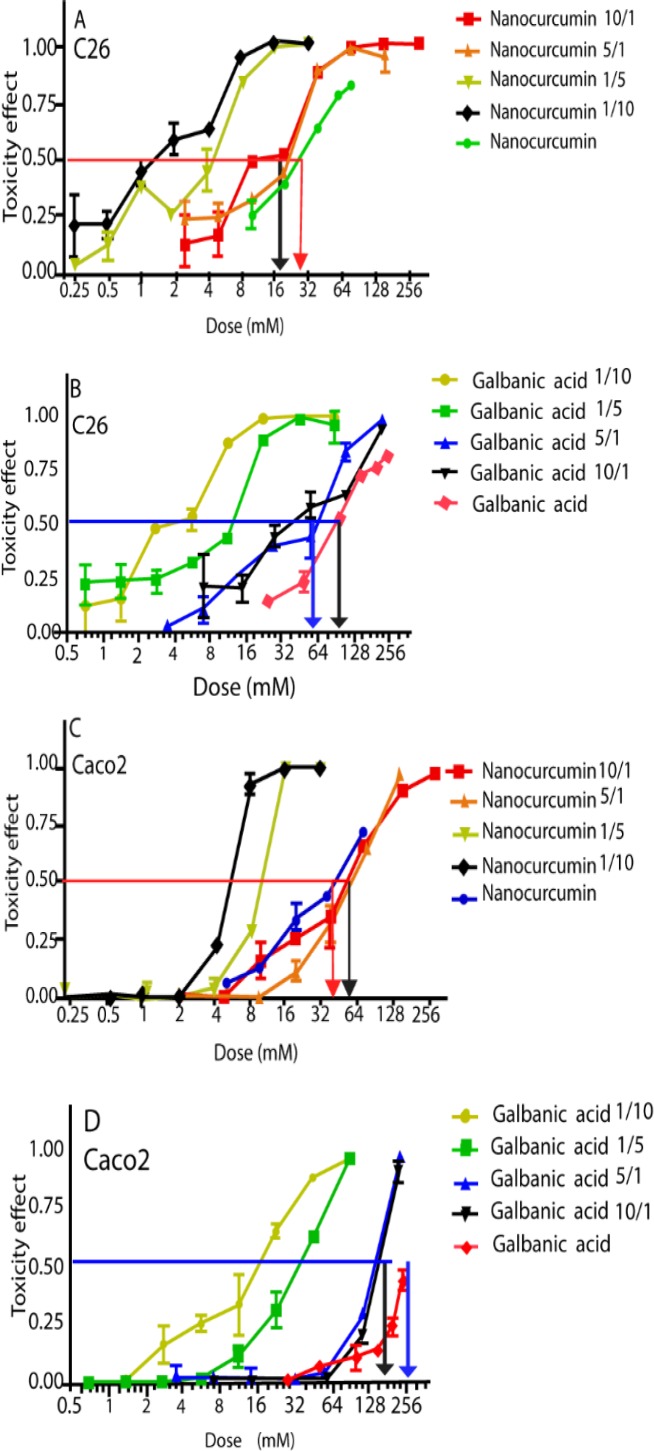
The dose-dependent cytotoxicity of the nanocurcumin and galbanic acid as well as their combinations at varying dose ratios. The 50% toxicity effect, i.e. horizontal line, is given. The black vertical arrows show the least IC_50_ values of nanocurcumin or galbanic acid in the combination. The red and blue arrows show the IC_50_ values of nanocurcumin and galbanic acid alone, respectively. Data are shown as mean±SD of 8 replicates (p<0.05)

Figure 3 shows the additive impact of curcumin plus galbanic acid on the cancer cell survival as compared to the nanomicellar curcumin and galbanic acid alone. The addition of the nanomicellar curcumin to galbanic acid shifted the dose-response curve to the left compared to treatment with nanomicellar curcumin alone (Figures 3A and C). The dose-response curve was also shifted to the left compared to treatment with galbanic acid alone (Figures 3B and D). All curves related to the combined treatments were positioned on the left side of the reference curves associated to the treatment of nanomicellar curcumin (red arrow on Figure 3A) and galbanic acid (blue arrow on Figure 3B). However, the curves relating to the nanomicellar curcumin 10/1 (denoting 10-to-1 dose ratio of nanomicellar curcumin to galbanic acid) and nanomicellar curcumin 5/1 treatments were positioned on the right side of the nanomicellar curcumin reference curve (Figure 3C). All curves related to the combined treatments were positioned on the left side of the reference curve of galbanic acid treatment (Figure 3D). 

In C26 cells, the nanomicellar curcumin/galbanic acid combination therapy led to a synergistic cytotoxicity response at the three inhibitory effective doses of ED50, ED75 and ED90 (Given CI<1). The combination therapy also led to a reduced required dose for both nanomicellar curcumin and galbanic acid (Given drug reduced index (DRI) >1) (Table 1). 

In Caco-2 cells, however, the nanomicellar curcumin/galbanic acid combination therapy showed additive and somehow antagonistic drug-drug interactive cytotoxic responses at nanomicellar curcumin/galbanic acid ratios of 10:1 and 5:1 (CI=>1). As a result, no reduced required dose was obtained using the combination therapy (DRI=1) (Table 1).

**Figure 4:**
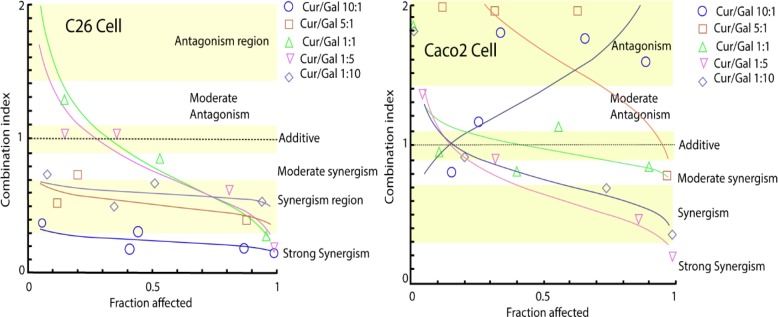
The combination indexes at varying concentrations and varying nanocurcumin (Cur): galbanic acid (Gal) ratios for C26 and Caco-2 cancer cells. The regions for different interactive responses (antagonism, additive, and synergism) are shown with the alternate yellow and white colors. The horizontal axes show the cell proportion killed by the reagents doses from 0 (no cell killed) to 1 (all cells killed). The mean of the data is shown in the graphs

Figure 4 shows the CI values within the range of inhibitory responses from 0 fraction-affected to 1 fraction-affected, where the fraction-affected term denotes the fraction of the cell population that is killed in response to a given dose. For C26 cells, only at very low doses (0 to 0.3 fraction-affected), the CI values was more than 1 for the nanomicellar curcumin/galbanic acid combination at 1:1 and 1:5 dose ratios. When nanomicellar curcumin:galbanic acid ratio was above 1, all the CI values was less than 1. On the other hand, for the Caco-2 cell, the nanomicellar curcumin/galbanic acid combination exerted an antagonistic inhibitory effect on the cell viability at 10:1 and 5:1 dose ratios (CI>1). The calculated CI values were less than 1 for the nanomicellar curcumin/galbanic acid combination at other dose ratios (Table 1).

**Nanomicellar curcumin combination therapy with some common therapeutics**

**Figure 5:**
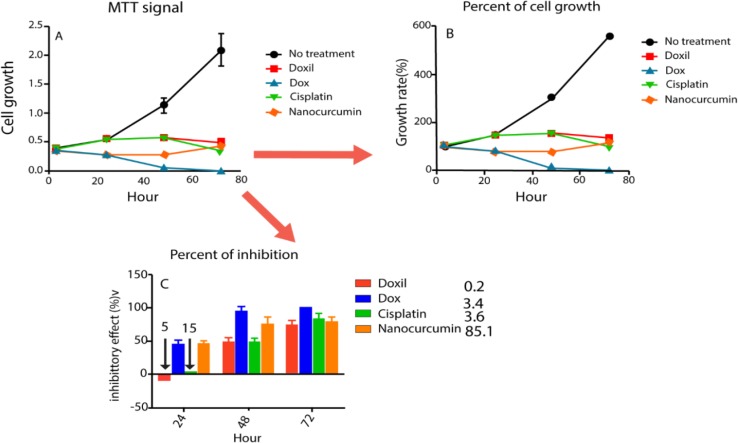
Time-evolution cytotoxicity of nanocurcumin and some common therapeutics. A shows the cell growth profile of the C26 cells in terms of MTT color development in the wells; B shows the normalized data in terms of the percent of cell growth taking the MTT absorbance of the wells 6 hr post-cell inoculation as 100%; and C shows the percentage of inhibitory effect of the tested drugs and formulations, taking the control drug-untreated wells as 100. C legend shows the drug doses (µM) relating to the inhibitory effect within 72 hr treatment. For Doxil and cisplatin, even the extremely high doses of 5 and 15 µM, respectively, showed no cytotoxicity at 24 hr. Data are shown as mean ± SD (n = 4)

Treatment of C26 cells with Doxil and cisplatin revealed that they were nontoxic during the first 24h of exposure to the cancer cells (Figure 5); Whereas the Dox and nanomicellar curcumin caused cancer cell growth inhibition following 24h treatment at high doses (Figure 5C). With time, the inhibitory impact of similar doses of the tested agents was more pronounced as these agents inhibited cancer cell growth completely (Figures 5A and B). Dox killed virtually all the cancer cells within 72hr, while it was only half-effective in killing cancer cells at the same dose 24 hr post-treatment. 

Combination therapy with nanomicellar curcumin also showed interesting results. Nanomicellar curcumin/Doxil and nanomicellar curcumin/cisplatin combination therapy did not result in a significant drug-drug interactive response at low doses of the nanomicellar curcumin (non-significant CI values at Doxil/Cur 10:1 and cisplatin/Cur 10:1 are shown in Table 2). However, a synergistic cancer cell growth limitation (CI<1) was observed at the high dose ratios of the nanomicellar curcumin (at Doxil/Cur 1:10 and cisplatin/Cur 1:1 ratios). 

**Table 1:** The parameters relating to the interactive inhibitory response of the nanomicellar curcumin/galbanic acid combined therapy 48 hr post-treatment

C26 colon carcinoma cell line						
Nanocurcumin-Galbanic acid	CI values at inhibition of			DRI values at inhibition of		
	50%	75%	90%	50%	75%	90%
10:1	0.53 ± 0.09^1^	0.42 ± 0.04	0.34 ± 0.01	2.1: 20.4^2^	2.6: 27.7	3.2: 36.1
5:1	0.68 ± 0.07	0.64 ± 0.01	0.62 ± 0.04	1.8: 8.4	1.8: 9.5	2.0: 11.5
1:1	± 0.05	0.70 ± 0.03	0.48 ± 0.02	2.2: 1.8	3.0: 2.7	4.2: 4.1
1:5	0.80 ± 0.08	0.58 ± 0.06	0.42 ± 0.05	7.9: 1.5	10.2: 2.1	13.0: 3.0
1:10	0.81 ± 0.18	0.74 ± 0.09	0.68 ± 0.01	16.9: 1.4	16.6: 1.5	16.4: 1.6
Caco-2 human colon carcinoma cell line						
Nanocurcumin-Galbanic acid	CI values at inhibition of			DRI values at inhibition of		
	50%	75%	90%	50%	75%	90%
10:1	1.08 ± 0.24	0.95 ± 0.16	0.84 ± 0.09	1.0: 27.7	1.1: 22.9	1.3: 19.0
5:1	1.34 ± 0.17	0.87 ± 0.10	0.57 ± 0.06^3^	0.8: 11.1	1.2: 12.6	2.0: 14.4
1:1	0.87 ± 0.04	0.77 ± 0.04	0.70 ± 0.04	1.6: 3.9	2.0: 3.5	2.6: 3.2
1:5	0.54 ± 0.04^3^	0.35 ± 0.01^3^	0.22 ± 0.03	5.1: 2.9	10.1^4^: 4.1	19.8^4^: 5.9
1:10	0.72 ± 0.12^3^	0.51 ± 0.08^3^	0.36 ± 0.06^3^	7.3^4^: 1.7	13.4^4^: 2.3	24.7^4^: 3.1

1 . Shows significant difference as compared to the CI (combination index) values of nanocurcumin-galbanic acid at 1:1 dose ratio (p<0.05).

2 . The right and left Figures in the datasets show the DRI (drug-reduced index) values for nanocurcumin and galbanic acid, respectively.

3 . Shows significant difference as compared to the CI values of nanocurcumin-galbanic acid at 10:1 dose ratio (p<0.05).

4 . Shows significant difference as compared to the DRI values of nanocurcumin-galbanic acid at 10:1 dose ratio (p<0.05).

**Table 2:** The parameters related to the inhibitory response of the drugs alone and in combination with the nanomicellar curcumin 48 hr post-treatment

	Drug dose at inhibition of				
	50%	75%		90%	
Doxil	0.23	1.03		4.48	
Nanocurcumin	53.5	84.9		134.8	
Dox	0.08	0.33		1.32	
Cisplatin	3.56	12.70		28.50	
	CI values at inhibition of				
	50%		75%		90%
Doxil/Cur (10:1)	ns^1^		ns		ns
Doxil/Cur (1:10)	0.58		0.40		0.31
Cisplatin/Cur (10:1)	ns^2^		ns		ns
Cisplatin/Cur (1/1)	0.68		0.73		0.84

Dox: doxorubicin; Cur: curcumin; CI: combination index.

1 . Shows non-significant difference as compared to Doxil (pegylated liposome doxorubicin) alone (p<0.05).

2 . Shows non-significant difference as compared to cisplatin alone (p<0.05).

## Discussion

It was of high importance to evaluate the drug-drug interaction in eliciting the cytotoxic response. In this process, we applied varying dose ratios of nanomicellar curcumin/galbanic acid to evaluate the impact of the combined treatment of these phytochemicals on the cells’ growth. The addition of the nanomicellar curcumin to galbanic acid shifted the dose-response curve toward left, meaning that the cell toxicity of the nanomicellar curcumin and galbanic acid increases when the other agent is added (Figure 3).

The nanomicellar curcumin/galbanic acid combination displayed no antagonistic anti-cancer activity in C26 cell, given that all the curves related to the combined treatments were positioned on the left side of the reference curves associated to the treatment of nanomicellar curcumin (red arrow on Figure 3A) and the galbanic acid (blue arrow on Figure 3B). However, the nanocurcumin and galbanic acid treatment exerted some antagonistic cytotoxic effects in Caco-2 cells since the curves relating to the nanomicellar curcumin 10/1 (denoting 10-to-1 dose ratio of nanomicellar curcumin to galbanic acid) and nanomicellar curcumin 5/1 treatments were positioned on the right side of the nanomicellar curcumin reference curve (Figure 3C). As nanomicellar curcumin was more toxic to the cells compared to galbanic acid, the cytotoxicity was more likely to be attributed to nanomicellar curcumin in combination compared to galbanic acid.

To determine the mode of nanomicellar curcumin and galbanic acid interactive responses, the data gathered from these experiments were compared with the data obtained from the section “Cytotoxicity assessment of single agents” using CompuSyn software. The parameters related to the combined treatment showed a synergistic drug-drug interactive response. CI shows the interactive relationship between two or more drugs in eliciting a given response (Chou, 2006), which is calculated as follows:

$\mathrm{CI}=\frac{D_{A:A+B}}{D_{A}}+\frac{D_{B:A+B}}{D_{B}}$ + …

Where D_A:A+B_ shows the dose of the drug A in combination at a given response to the dose of the drug A (D_A_) alone at that response. Similarly, D_B:A+B _shows the dose of the drug B in combination to the dose of the drug B (D_B_) alone. According to the equation, CI=1 represents no drug interactive response. In other words, it indicates that an additive relationship exists between two or more drugs in eliciting a particular response. A CI>1 indicates an antagonistic effect and a CI< 1 indicates a synergistic effect (Chou, 2006 ▶; Zhao et al., 2004 ▶). 

In C26 cells, the CI values at the three inhibitory effective doses of ED50, ED75 and ED90 were all below 1, indicating that the nanomicellar curcumin/galbanic acid combination therapy exerts a synergistic cytotoxic effect on the cells. DRI shows the dose of the drug alone at a given effect (for instance ED50) divided by the dose of that drug in the combination to produce the same effect. For C26 cells, all DRI values for the inhibitory effect of the nanomicellar curcumin and galbanic acid was > 1, indicating that the combination reduces the required dose for both agents to elicit particular inhibitory responses (ED50, ED75 and ED90). According to the CI and DRI values for Caco-2 cells, it was found that nanomicellar curcumin/galbanic acid combination therapy exerts no synergistic inhibitory effect on the cancer cell growth of this specific type of cancer cell. 

As both curcumin and galbanic acid fell far short of enough cancer cell growth limitation at low doses, which is presumed to be achieved *in vivo*, it was much more realistic to assess the cancer cell inhibitory activity of the nanomicellar curcumin combined with some common chemotherapeutics such as Dox and cisplatin. These agents are putatively approved to have significant anticancer effects (Leibbrandt and Wolfgang, 1995 ▶; Barenholz, 2012 ▶). Since galbanic acid was found to have limited cytotoxicity and poor water solubility, it would be far-fetched to be regarded as a promising anticancer agent. For galbanic acid to become a truly anticancer agent, very high doses are theoretically needed to reach tumor region following intravenous injections, which would be impossible given the poor water solubility of the agent. As a result, galbanic acid was disregarded for the combination therapy. 

We conducted the 24-hr combination therapy using the nanomicellar curcumin and the chemo-agents and no synergistic interactive cell inhibitory effect was observed at any of the mentioned combinations and dose ratios. In fact, treatment of C26 cells with Dox, Doxil and cisplatin revealed that they have become more toxic to the cells with increasing time of exposure to the cells. 

Considering different time-evolution cytotoxicity profiles of the agents, nanomicellar curcumin is supposed to exert optimum synergistic effect if administered 24 hr after Doxil and cisplatin injection. To study this hypothesis, nanomicellar curcumin was applied in combination with Doxil at 1-to-10 and 10-to-1 of curcumin:dox dose ratios. Similarly, it was applied in combination with cisplatin at 1-to-1 and 1-to-10 dose ratios. These combination therapies led to an improved anti-cancer effects when nanomicellar curcumin was used at high doses as compared to those of Doxil and cisplatin. This means that Doxil and cisplatin cancer chemotherapy could benefit from post-treatment with nanomicellar curcumin, when the dose of the chemo-agents reduces. 

Taken together, it was found that the nanomicellar curcumin and galbanic acid, two phytochemicals with anti-cancer properties, exerted a synergistic cell growth inhibition in the cancer cells. However, this effect was dose- and cell type-dependent. High dose ratios of nanomicellar curcumin:galbanic acid were found to produce higher inhibition of cancer cell growth compared to low dose ratios ,in C26 cancer cells. However, in Caco-2 cells, low dose ratios of nanomicellar curcumin:galbanic acid more effectively inhibited cancer cell growth. Although we could not explain why varying nanomicellar curcumin:galbanic acid dose ratios elicited different interactive responses in different cell types, we could provide a recipe for an optimum cancer treatment using a combination of nanomicellar curcumin and galbanic acid. Whether or not they could improve cancer therapy in combination with the current chemotherapeutics like Dox and/or cisplatin in cancer-bearing model animals merits further investigation to clarify the pharmacokinetic profile, cell uptake potential, and cell toxicity of these agents in normal cells.
